# Barriers to the composition and implementation of advance directives in oncology: a literature review

**DOI:** 10.3332/ecancer.2019.974

**Published:** 2019-11-12

**Authors:** Pedro Grachinski Buiar, José Roberto Goldim

**Affiliations:** 1 Medical Oncology Department, Hospital de Clínicas de Porto Alegre (HCPA), Porto Alegre, RS 90035-007, Brazil; 2 Bioethics Division, Hospital de Clínicas de Porto Alegre (HCPA), Porto Alegre, RS 90035-007, Brazil; a http://orcid.org/0000-0001-5144-1197; b http://orcid.org/0000-0003-2127-6594

**Keywords:** advance directive, cancer, neoplasm, factors, palliative, living wills

## Abstract

The advance directive (AD) is an important resource in oncology and all areas of medicine directly involved in the care of palliative patients. It provides people with the right to have their living wills honoured when they cannot respond by themselves. Despite their importance, ADs are still underused in most countries due to multiple factors. The objective of this review is to better categorise the barriers and difficulties that could impair the composition and implementation of ADs, allowing direct efforts against these obstacles. After the literature review, we believe that there would be five steps in the trajectory of an AD (discussion, composition, registration, access and implementation) and that all those steps can be affected by factors involving the health systems and professionals, the patient themselves and relatives or caregivers.

## Introduction

**Figure 1.**
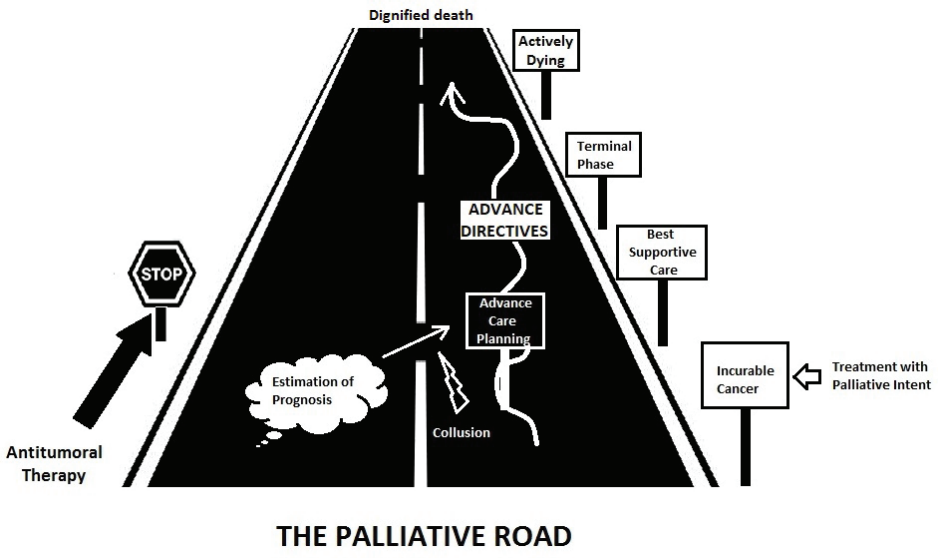
‘The palliative road’ illustrates the trajectory of the patient in palliative treatment since the diagnosis of the incurable disease until the death event, going through all the steps marked in the evolutionary history of the disease. Ideally, ADs should arise from the beginning and accompany the patient through the entire trajectory contributing to a good death in the end.

Advances in cancer treatment have increased the number of patients with prolonged survival [1]. One major threat in modern oncology is the excessive focus on treatment response rates and the tendency to neglect research and studies about the care of dying people [2]. The concept of patient-centred care is built on patient preferences and beliefs [3]. And those should be readily available in the form of advance directives (ADs), one of the products derived from the Advance Care Planning process which should be started at the moment of an incurable diagnosis and maintained throughout the palliative trajectory (Figure 1) of the cancer patient [4]. An AD is commonly manifested by two documents, the Living will (that is directly related to the patient-specific preferences, regarding the medical care they want to receive, including preferences like place of death, life-sustaining treatments and any other points of treatment or care that are important to the person) and the Power of Attorney (a document that enables another person to decide on the name and to handle questions that an incapable person leaves unresolved).

ADs contribute to the attenuation of suffering (of patient and family) and anxiety, to the augmentation of autonomy, psychological relief and patient satisfaction [5]. Health systems are benefited too, with a reduction in unnecessary costs [6–9].

Despite the benefits referred to above, the implementation rates of ADs vary widely in the literature. A recent systematic review, analysing 795,909 people in the United States, indicated a stable percentage of around 36.7% of adults having ADs, and 29.3% were characterized as living wills [10]. Restricting the analysis to patients with cancer, this number reaches a rate of nearly 70% [11].

ADs are derived from the Advance Care Plan (ACP), which is not a concrete object but a process developed over time and constructed through shared decision-making. This process could also be impaired by a lot of factors such as lack of experience by physicians, absence of adequate protocols, difficulty in estimating prognosis and providing information, difficulty in talking about death and making decisions about future scenarios, slow decision-making capacity by patients, low level of concordance between the patient’s wishes and family opinion and many other factors [12–15].

The main objective of this study is to review the major obstacles making it difficult for palliative patients to exert full autonomy, achieving a ‘good death’ by having their beliefs and wishes respected.

## Methods

We performed a literature review using the PubMed database looking to respond to the question ‘What are the barriers and difficulties preventing the advance directives being successful?’. The descriptors used were ‘Advance Care Planning’, ‘Advance Directives’, ‘Cancer’, ‘Oncologic *’ and ‘Palliative’. Articles published in the past 40 years were considered, obtaining a total of 1,227 articles. After excluding articles of editorials, paediatric cancer patients, noncancerous patients with dementia and articles not published in the English language, we reviewed 121 articles and selected 81 for this review paper composition. The final search was performed in June 2019.

## Results

### Health system-related factors

Early discussions about advanced care should be initiated when patients are in outpatient/office-based care. In a stable clinical period, with adequate time and place for reflection, people have the most appropriate conditions for discussion and review of directives. It must be clarified to the patient and family that ACP and ADs are not a ‘one-time event’ [16]. It can be reviewed and replaced at each visit or whenever a new event changes the patient condition or prognosis. A cohort study by Mack and colleagues demonstrated an inverse association between end-of-life (EoL) discussions before the last 30 days of life and the application of aggressive measures [17]. The ability to prognosticate correctly is involved in the aggressiveness of EoL care in patients with metastatic cancer receiving palliative therapy. Patients receiving an incorrect life prediction are more likely to suffer aggressive EoL care (OR 2.55; 95% CI, 1.09–5.99; *p* = 0.03) [18].

It is not uncommon to see health professionals believing that EoL discussions will lead patients to higher levels of stress and discomfort. Evidence shows that up to 98% of patients with less than 6 months of life expectancy want realistic information from their physicians, but 91% of these patients report that the physician is uncomfortable or nervous with this type of conversation [19]. In another study that evaluated patients desiring to be informed about prognosis, only half of them were informed about life expectancy [20]. Finally, it is also important to bear in mind that the majority of patients do not complain about additional depression/anxiety after discussions about prognosis. Actually, the lack of prognostic information is considered a real complaint regarding EoL care [9, 21, 22]. This fear of addressing bad news and discussing ACPs and ADs on the part of doctors and health professionals should be mitigated in the light of the current evidence.

If we look at physician behaviour when caring for cancer patients, we see that only 18% of doctors talk about do-not-resuscitate (DNR) status promptly versus 26% that wait until there is no more antitumour active treatment available. Considering the conversation about palliative care enrolment, the percentage of doctors that postpone this discussion until there are no more available treatments rises to 49%. In the multivariable analyses, one factor associated with earlier talks about hospices was the office-based practice (*p* = 0.007). Another interesting aspect is that the oncologist is the specialist that discusses prognosis more frequently, but the non-cancer specialist is responsible for the majority of early discussions about DNR code, hospice enrolment and site of death (*p* < 0.001) [23]. Although the general population asserts that the diagnosis of advanced and incurable cancer is reason enough to trigger this discussion between family and physician, most health professionals say they do not initiate EoL discussions with patients who are feeling well but wait until they become symptomatic or there are no more available antitumour treatments. This fact may explain why 75% of patients want their physicians to start discussions about EoL and ACPs but only 5% of doctors start this task in some retrospective series [24, 25]. When we look at the oncologists’ performance, half of them contribute to their patient’s ADs [26].

A personal history of palliative care consultation elevates the odds of advance care planning documentation (OR 6.79, *p* ≤ 0.001) [27] and the different global availability of palliative care referral could be a reason for disparities in the literature [28].

Even after they have been created and registered, ADs might be not followed. A study published by Biola *et al* [29] found that 89.1% of the relatives of terminally ill patients reported a decision on resuscitation, 82.1% on artificial enteral nutrition, 64.3% on the administration of antibiotics and 83.7% on transfer to hospitals. Paradoxically, the directive was not followed by the medical team in 71.4% of the resuscitations, 14.3% of the enteric tubes, 19.2% of antibiotics and 29.9% of the hospital transfers. One possible cause for this might be a loss of information during the transition between different sites and teams of medical assistance, for example, from home to emergency rooms. The emergency departments are visited by a lot of cancer patients, and patients in terminal conditions routinely receive some type of invasive life-sustaining measure [30, 31]. Emergency departments are not the best place for difficult conversations and complex decision-making regarding discontinuation/abstention of therapies [32]. For this reason, ADs should be readily available and documented in some type of portable device [33]. An instrument developed trying to mitigate this problem is called Physician Orders for Life-Sustaining Treatment (POLST). POLST is an EoL care transition programme that focuses on patient-centred goals and that informs shared decision-making directives. It provides a mechanism to communicate the desires of severely ill patients regarding medical treatments to be instituted or avoided in transition between health institutions and health teams. The objective is to standardise and make the living wills prevail over any opinion, conduct or focus that may vary among different health institutions or professionals across the patient trajectory [34]. Despite this, mechanisms relying on physical documents are susceptible to losses and problems of visualisation/interpretation. One way to overcome this barrier is the use of a shared electronic health system to register and deliver medical information about patient decisions in EoL. But an electronic health registry needs to be shared between health facilities, to be organised centrally and also needs to provide training and stimulus for the documentation of goals of care [35].

Recently, a randomised clinical trial concluded that a communication quality-improvement intervention (that included clinician and patient tools, clinician training and some practical systems changes) could raise the rates of documented discussions about preferences, goals and prognosis (96% versus 79%) and found that those discussions occurred earlier when compared with the control group (median of 2.4 months; *p* < 0.001). However, despite the positive effect regarding conversations, the effective documentation of EoL plans did not reach a statically significant difference [36]. This could be explained by the influence of multiples factors involved with the ADs and the ACP.

Finally, even apparently simple and unexpected details such as a correct palliative International Classification of Diseases 10th edition code and a primary care connection could prevent unnecessary emergency visits [37].

### Patient-related factors

Demographic data associated with the higher rates of ADs registration include being Caucasian, high educational level, high economy category, residents of nursing homes and subjects with a neoplasm diagnosis [38]. The decisional patterns regarding patient preferences vary around the globe, from active to passive models, like those encountered in Singapore, South Africa and Brazil [39].

One important modifiable factor involved in the composition of ADs is the lack of knowledge/poor understanding regarding illness, which may be attributed to patient-related lack of interest or to physician’s omission [40]. A study involving patients with stage IV lung and colorectal disease who received palliative chemotherapy showed that only 33% of them recognised the chemotherapy as not curative. This group of patients was more likely to enrol in hospice care and also to receive less aggressive measures (OR 1.97, 95% CI=1.26–2.66) [41].

Another main reason patients fail to complete their directives is the difficulty in anticipating their wills based on scenario projections and specifying preferences or limitations for life-sustaining therapies, a fact that can be attenuated by the presence and help from a medical professional during the discussion of directives and document composition [5, 24, 42]. Ideally, this professional should be the long-term assistant physician.

One way to overcome these barriers related to scenario projection is the use of complementary instruction mechanisms like educational videos, something that gives more power to the patient in the decision process [43].

Discussions about EoL are one of the most difficult conversations in all medicine. Eighty-four percent of patients would like to discuss treatment symptoms and toxic effects at the time of diagnosis of metastatic disease, and 59% of them would like to discuss life expectancy at the same moment [44]. At some point, the patient needs to know their prognosis since those who do not know usually tend to prefer more aggressive treatments [45]. Nowadays, it is acceptable to have some uncertainty around prognostication with the addition of new treatments and clinical trials enrolment. We must bear in mind that these new treatment results were not included in the majority of classical prognostic models and nomograms [46].

When we look at metastatic subjects who have already decided, we see that only 39.5% have a preference for non-resuscitation but only half of those had their orders for non-resuscitation documented. It is imperative to think about the reasons for this number [47].

A study evaluating 2,538 terminal cancer subjects admitted to the Palliative Care Unit of a Cancer Centre with a clear medical indication of non-resuscitation found a 4% rate of non-resuscitation order refusal by those patients. The factors associated with this behaviour are poor analgesic control (*p* = 0.0005), presence of nausea (*p* = 0.05) and dyspnoea (*p* = 0.002). After multivariate analysis, the association is with moderate to severe pain (OR 3.19, *p* = 0.002) and the absence of registered living wills (OR 2.94, *p* = 0.001) [48]. This data illustrates the importance of the management of factors at the patient level, like adequate symptoms control and higher quality of life provision.

Another problem in composing a directive may be the lack of time due to the rapid evolution of the disease accompanied by cognitive impairment and worsening of the general health condition. A large retrospective study with more than 200,000 cases show that the median interval (in days) between the DNR order signature and the patient death was 0 days, i.e., on the same day, the patient had died. On subgroup analysis, subjects in the outpatient scenario had a median interval of 30 days between DNR status signature and death [49].

This may illustrate the tendency of the decision-making process to occur closer to death. We know that cancer patients have an abrupt decline of their functional capacity in their last 3 months of life and that the cognitive decision-making capacity in terminal patients tends to be more limited [50]. The ability to express a choice is preserved, but the understanding, processing and reasoning capacity seem to be impaired [51]. Despite guidelines recommending ADs discussions occur as soon as possible, decisions like DNR are still occurring late or very close to the death event [47, 52–55].

The amount of time dispensed to ACP and EoL discussions in an office-based consult are important. Approximately 90% of patients exposed to some forms of ADs have not completed these documents and say they still needed additional information and time to take some of the decisions [56]. As a result, this task frequently ends in the hands of a relative/caregiver.

### Caregiver and relative-related factors

The family plays a central role in the support of the patient near to the EoL and in the decision-making process [57, 58]. Despite being recognised by the health team as representatives of the patients, it is common that those relatives have not been formally designated by the patients themselves as attorneys. And those people, given the burden of responsibility and feelings like guilt or regret, may choose to maximise the patient’s life, no matter how iatrogenic it may be.

A 12-year prospective study analysing changes in the prevalence rate of the ACP followed patients from 2000 to 2012 and found that despite a significant increase in the Durable Power of Attorney rate (52%–74%), the rates of living wills (40%) and discussions with patients about EoL questions (60%) did not change [59]. Clinicians need to bear in mind that the results from a discussion about EoL with relatives may be too distant from the results of the same discussion with the patient himself.

In paternalistic cultures, where the family assumes the responsibility of decision-making regarding the patient’s EoL, we can witness the phenomenon called ‘collusion’—behaviour characterised by hiding the truth from patients with the tendency to make decisions without including them in the process and discussing with third parties. Some studies indicate that about 95.2% of registered ADs were actually defined by the family members in charge, and not by the patient themselves [11]. Other data showed up to 87% of prognoses are first revealed to a family member of a cancer patient [19]. One potential detrimental effect resulting from decisions taken by family members is the possible divergence of opinions between the family member and the patient. Especially when there was insufficient time for that family member to reflect and think about the patient’s preferences in life [60]. It is very important to reinforce that the directives should be ideally created by the patient themselves, as personal autonomy is one of the key ethical principles of modern medicine.

One way to alleviate the potential suffering and pressure of family members responsible for EoL decisions would be to extend the interval between the beginning of these discussions and the date of the patient’s death [61, 62]. There are also some data that show that when requested early and under adequate conditions, the involvement of the relative is associated with increased patient satisfaction and reduced levels of depression and anguish [63]. The simple fact of being nominated as a legal representative previously to the outcomes of the ill patient could also alleviate the psychological charge in the family members [64].

One independent factor related to caregivers is their educational level. A study suggests that the desire for aggressive measures and life-sustaining treatments for the patient tends to be inversely proportional to the cognitive level of their caregivers [65].

We cannot ignore that even when finalised, ADs may fail to achieve their goals when the patient and family members do not understand how they will be used or their goal. It is estimated that 28%–30% of patients did not discuss their AD with family members for undefined reasons. The same data also revealed that up to 91% of these patients did not discuss their ADs with the attending physicians [66,67]. Every health professional should be alert and actively question the patient about living wills.

Finally, we should keep in mind the implications for caregivers and relatives who must take the decision in the name of the patient. A study with patients who had died in intensive care units revealed that the rate of post-traumatic symptoms in the relatives who took direct decisions for the patient was 82% compared to 60% in the relatives whose decision was not required [68].

### Low quality of evidence regarding AD and life-sustaining measures

There is a lack of randomised controlled studies to assess the benefit of life-sustaining measures in the palliative scenario. A Korean study evaluating decisions of terminal patients classified the life-sustaining treatments into two types: General and special. General measures were considered to be enteral feeding tubes, oxygen therapy and laxatives and special treatments were considered to be cardiopulmonary resuscitation, mechanical ventilation, dialysis, blood components transfusions, chemotherapy and wide-spectrum antibiotics [11].

The concept of ‘futility of a treatment’ is defined quantitatively, by the probability of therapeutic success, and qualitatively, by the value conceived in terms of quality of life [32]. A futile treatment is characterised by the absence of benefits, or by the disproportion between benefits and associated risks. Health Practitioners should always keep in mind that the value of small gains in overall survival is extremely controversial. Seven days could mean everything for a patient that has some degree of life and social interaction or could mean nothing to a patient with no interaction, in suffering or bedbound. An appropriate ACP constructed through a shared decision-making process with the composition of an AD may be the most appropriate tool to guide difficult health decisions.

Artificial nutrition in oncology is prescribed mostly to maintain nutrition support in patients unable to eat during the active antitumour treatment, either due to cancer- or treatment-related effects. Artificial nutrition in patients with limited prognosis does not seem to change outcomes in the course of advanced disease [69]. Parenteral hydration also seems to have limited value to prolong survival, in this case with a randomised placebo-controlled trial demonstrating no difference in median survival, although this trial had not obtained sufficient statistical power (*p* = 0.83) [70]. Regarding cardiopulmonary resuscitation (CPR) in cancer patients, a meta-analysis of 42 studies comprising 1,707 cancer patients submitted to an in-hospital CPR concluded that the overall survival rate was 5.6% for metastatic patients and 9.5% for patients with localised disease [71]. A large cohort study published after this meta-analysis compared the resuscitation outcomes of patients with and without advanced cancer. Cancer patients achieved a 7.4% rate of survival to discharge compared with 13.4% of matched subjects without cancer (*p* < 0.001) [72].

Blood components transfusion in terminally ill cancer patients is another question for debate. A Cochrane Review evaluating blood transfusions in cancer patients found no randomised clinical trials and concluded that 31%–70% of patients with advanced cancer showed some degree of improvement in breathlessness, fatigue and general well-being. This review also found that 35% of patients die within 2 weeks after the palliative transfusion [73]. A retrospective study analysed 309 dying patients with terminal cancer between 2010 and 2011 and compared the survival outcome of patients that received blood transfusion versus controls without transfusions. This study found that 90% of patients had anaemia during their last hospitalisation and 38.4% received red blood cells (RBC) transfusion. The median survival in the transfusion group was 15 days versus 8 days in the group with no RBC transfusion (*p* < 0.001) [74].

Unwanted and unnecessary hospitalisations are routine in the disease evolution of terminal cancer patients, and several studies demonstrated that the main reason for this type of hospital admission is for symptom control [75–79]. The main complications and causes of death in cancer patients are sepsis, respiratory obstruction, intestinal obstruction, spinal cord compression and thrombosis [80]. It is mandatory to distinguish symptoms of easy palliation from scenarios where symptomatic relief necessarily depends on the reversal of those severe cancer-related complications, requiring aggressive procedures [81]. The question that arises is the actual magnitude of benefit from those interventions and the patient’s willingness to receive such interventions, with a potential risk for death without guarantee of symptomatic control, life prolongation or improvement in the quality of life.

## Conclusion

**Figure 2.**
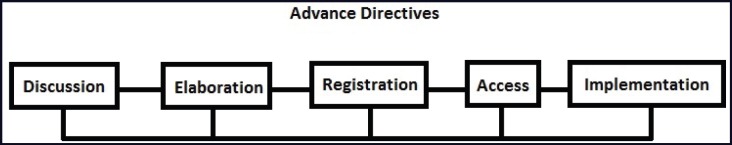
Five steps for a successfully implemented AD.

The major goal of the advance care planning process and ADs is to give the patient the right to a dignified death at the end of their life. This review inferred that there are a lot of factors that could affect the ACP process and ADs in a positive or negative manner, in all their phases (discussion, composition, registration, access and implementation—Figure 2).

**Figure 3.**
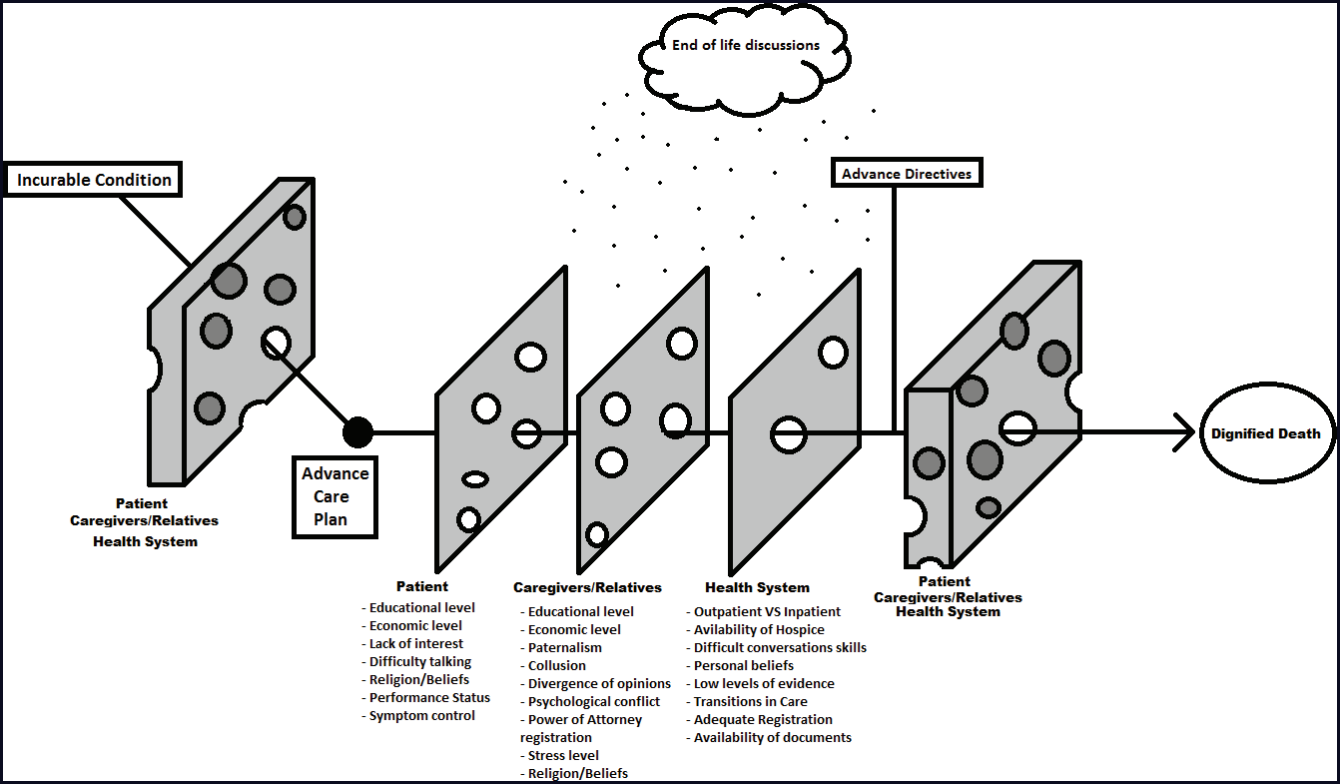
The multiple obstacles and barriers that ACP and ADs must pass through to guide the patient to their desired end.

We can divide those factors into non-modifiable and modifiable, and distribute them by steps, scenarios and people involved in the process of AD creation. Making an analogy with the ‘Swiss cheese model’ [82], we could say that the ACP and ADs must overcome a series of steps and multifactorial obstacles to be successful in their final objective (Figure 3). All medical specialists involved in the care of cancer patients with incurable conditions should pay attention to these factors and possible barriers, especially those potentially modifiable and directly influenced by our practice. An AD elaborated by the patient themselves, and that anticipates all the possible conflicts, is the best tool to make the right decision, and it is imperative to have in mind that after created, the AD should not be destroyed, forgotten or underused.

In the era of innovative and promising treatments, the medical practitioner should keep in mind that no matter how long the journey of life, the only certainty is the end. Some patients need aggressive treatments and others do not, some have opportunities to enter clinical trials and others do not and some have greater survival than others. Each palliative patient has a different trajectory. However, the common factor for all is the desire for a dignified death.

## Conflicts of interest

The authors declare that they have no conflicts of interest.

## Funding

All the authors declare no access to funding support.
